# [Expression of Concern] Insulin-dependent diabetes mellitus decreases osteoblastogenesis associated with the inhibition of Wnt signaling through increased expression of Sost and Dkk1 and inhibition of Akt activation

**DOI:** 10.3892/ijmm.2026.5799

**Published:** 2026-03-16

**Authors:** Mamiko Hie, Natsumi Iitsuka, Tomoyo Otsuka, Ikuyo Tsukamoto

Int J Mol Med 28: 455-462, 2011; DOI: 10.3892/ijmm.2011.697

Following the publication of this paper, it was drawn to the Editor's attention by a concerned reader that the TRAP-stained images shown in Fig. 1 for the 'Diabetes' and 'IGF-I' panels contained an overlapping section, such that data which were intended to have shown the results of differently performed experiments were apparently derived from the same original source.

The authors have been contacted by the Editorial Office to offer an explanation for the apparent anomaly in the presentation of the data in their paper, and we are awaiting their response. Owing to the fact that the Editorial Office has been made aware of potential issues surrounding the scientific integrity of this paper, we are issuing an Expression of Concern to notify readers of this potential problem while the Editorial Office continues to investigate this matter further.

